# Early Recognition of Behaviour Problems in Shelter Dogs by Monitoring them in their Kennels after Admission to a Shelter

**DOI:** 10.3390/ani9110875

**Published:** 2019-10-28

**Authors:** Liam Clay, Mandy Paterson, Pauleen Bennett, Gaille Perry, Clive Phillips

**Affiliations:** 1 Centre for Animal Welfare and Ethics, University of Queensland, Gatton, Queensland 4343, Australia; mpaterson@rspcaqld.org.au (M.P.); c.phillips@uq.edu.au (C.P.); 2 Royal Society for the Prevention of Cruelty to Animals Queensland, Brisbane, Queensland 4076, Australia; 3 School of Psychology and Public Health, La Trobe University, Bendigo, Victoria 3552, Australia; pauleen.bennett@latrobe.edu.au; 4 Delta Society, Summer Hill, New South Wales 2130, Australia; perrygaille@gmail.com

**Keywords:** behaviour, problems, assessment, canines, shelters, predict

## Abstract

**Simple Summary:**

Canine behaviour assessments are commonly used in shelters to identify behaviour problems in dogs prior to adoption. The aim of this study was to evaluate whether kennel monitoring of dogs could identify early signs of behaviour problems. Kennel behaviour was monitored for 38 dogs in their first five days in kennels at a shelter in Brisbane, Australia. This was compared to a formal assessment of exploratory, handling, play, run/freeze, and food guarding behaviour, as well as stranger and fake toddler interactions, and behaviour when the dog was alone, conducted five days after shelter admission. Kennel behaviours associated with fear, anxiety, and arousal in dogs were significantly correlated with the same behaviours in the formal assessment. With respect to outcomes, dogs that displayed more whining, tense body posture, standing leaning forward, panting, ears forward, less barking, lowered body and balanced/relaxed body posture, standing still, and standing by the wall had increased odds of failing the behaviour assessment. The study demonstrates that monitoring kennel behaviour could detect early signs of behaviour problems.

**Abstract:**

Canine behaviour assessments are commonly used in shelters to identify behaviour problems in dogs prior to adoption. The aim of this study was to evaluate whether kennel monitoring of dogs could identify early signs of behaviour problems, thereby facilitating early intervention and better management of dogs displaying behaviour problems. Kennel behaviour was monitored for dogs (*n* = 38) in their first five days in kennels at a shelter in Brisbane, Australia. This was compared to a formal assessment of exploratory, handling, play, run/freeze, and food guarding behaviour, as well as stranger and fake toddler interactions, and behaviour when the dog was alone, conducted five days after shelter admission. Kennel behaviours associated with fear, anxiety, and arousal in dogs were significantly correlated with the same behaviours in the formal assessment. Positional correlations were also evident. With respect to outcomes, dogs that displayed more whining, tense body posture, standing leaning forward, panting, ears forward, less barking, lowered body and balanced/relaxed body posture, standing still, and standing by the wall had increased odds of failing the behaviour assessment. Over the five days in the kennel, the frequency and duration of fear-related behaviours decreased, suggesting a reduction in arousal as the dog became accustomed to the shelter environment. The study demonstrates that monitoring kennel behaviour could detect early signs of behaviour problems.

## 1. Introduction

The largest Australian animal welfare organisation, the Royal Society for Prevention of Cruelty to Animals (RSPCA), received 40,286 surrendered dogs in the 12 months from July 2017 to June 2018 [1]. Reasons for dog relinquishment commonly include behaviour problems, e.g., inappropriate toileting, barking, digging, separation anxiety, fear, or aggression [2,3,4]. Entry to a novel shelter environment, plus alienation from its former owner, home, and routine, is likely to result in a potentially stressful form of social isolation in a surrendered dog [5]. Dogs experience fear and anxiety upon relinquishment to a shelter, with overt signs of stress sometimes persisting for several weeks after relinquishment [5,6]. Furthermore, as the length of time in a shelter increases, the detrimental impact on dogs’ emotional state worsens [7,8,9]. Coping capacity differs considerably between individual dogs, with variable habituation to the environment and the same stressor being experienced as neutral or aversive [10,11,12]. Therefore, in order to reliably and effectively assess and monitor the mental well-being of surrendered dogs, it is important that early interactions with the novel environment are recorded to identify signs of negative affect, e.g., separation anxiety, which occur with high frequency in adopted dogs from shelters [13].

Behaviour assessments are used in shelters globally, assessing adoption suitability, identifying behaviour problems, and matching dogs with the most suitable adoptees [14]. Veterinarians also implement a variety of testing procedures for quality of life assessments in animals with medical and behavioural issues [15]. However, behaviour assessments in shelters have been recently criticised, due to both the nature and consequences of pass or fail assessment procedures and doubt about their ability to accurately predict behaviour problems [16]. It is claimed that they cannot accurately determine the frequency of false positive (identification of a behavioural problem that does not really exist e.g., aggression, which renders the dog unfit for adoption) or false negatives (failure to detect a behavioural problem during the test). Usually, dogs are removed from their kennel to undertake the test in a standard facility, through which many other dogs have passed. This single context assessment is likely to present a stressful situation for the dog, which is unlikely to replicate the best environment to examine their anticipated behaviour in the home in which they are adopted. For example, the presence of excreta from previous dogs, or potentially even odours from dogs previously tested, can affect the outcome of tests [17].

An alternative is to observe behaviour in their kennel (hereafter kennel behaviour), handler interactions, and interspecies behaviour, allowing them to be tested in the environment into which they are becoming settled. Kennel monitoring has been used previously in shelters to identify behaviour problems [18,19,20]. Furthermore, kennel behaviour monitoring could potentially be automated, using for example motion sensing or by programming computers to recognise specific behaviour patterns, e.g., escape attempts [15].

There is a need for better observational tools for assessment in shelters [21]. These could include assessing behaviour longitudinally in shelters, to account for plasticity, and the greater predictability of behaviour when measured over a period of time [20]. Therefore, the aim of this study was to compare the manifestation of behaviours in a structured assessment with behaviours observed in their kennel over the first five days in a shelter.

## 2. Materials and Methods

### 2.1. Ethical Approval

This study was granted ethical approval from the University of Queensland Animal Ethics Committee (AE04214). All dogs were owner-surrendered, and permission was obtained from the owners to enrol their animals into the study.

### 2.2. Subjects

Criteria for dogs to enter the study were that they were between six months and 10 years of age, had no predisposed medical conditions and had not been previously admitted to the shelter. Thirty-eight dogs (18 male, 20 female) of mean age 3.1 years (SEM 0.37 years) and weight 20.3 kg (SEM 1.43) that had been surrendered to the RSPCA Queensland’s Animal Care Facility over a three month period were enrolled into the study. They represented the following 20 different breeds: Bull terrier (*n* = 9), Kelpie Cross (*n* = 6), Mastiff (*n* = 4), Beagle cross (*n* = 2), Staffordshire Bull Terrier (*n* = 2), and one each of Jack Russell cross, Alaskan Malamut, American Bulldog, Australian Cattle Dog, Australian Shepherd Cross, Border Collie, Boxer, Bull Arab cross, German Shepard cross, Husky cross, Labrador Retriever, Papillon, Poodle Cross, Portuguese Podengo, and Spoodle. All had been privately surrendered, with owners being required to declare the reasons for surrender.

### 2.3. Housing and Feeding

Dogs were housed in a single block of kennels, which held 16 dogs in individual kennels. Each kennel had a floor area of 3.5 m^2^ (120 cm × 180 cm), concrete floors and two solid walls separating each kennel and a gate opening into the kennel block, a fence opening out toward a garden area, a separate sleeping area with a raised bed, soft bedding, and toys. The dogs were fed twice daily with a combination of dry and wet food and had access to fresh water. Each dog received walks twice a day at 09:00 and 15:00 by shelter staff or volunteers.

### 2.4. Behaviour Monitoring

#### 2.4.1. Kennel

Dogs were observed on days 1–5, following surrender on day 1, for 60 min (07:30–08:30, before interactions with volunteers). Data were collected using two video surveillance cameras (KOBI CCD video cameras, Model: K-32HCVF, Taipei, Taiwan) placed in each individual kennel at a height of 3 m.

#### 2.4.2. Standard Behaviour Test

The standard RSPCA Qld behaviour assessment (RSPCA, 2018) was conducted on day 6, i.e., the day after the five days of kennel observations, as used by Queensland RSPCA shelters in each state to assess adoption suitability in shelter dogs. The assessment comprised a series of 10 tests of increasing provocation. Dog responses were scored based on frequency and durations of a variety of behaviours as described below. The tests were performed over 15 min with the following aids: a 1.8 m leash, tennis ball, plush squeaky toy, rope, plastic hand on a extend pole, bowl, raw hide or bone, and combination of wet and dry dog food.

The assessments were performed in a room (3 × 5 m) 20–30 m from the kennels, with one window and two half frosted doors, and a concrete floor with hospital-grade non-slip painted covering. All dogs were moved on lead from their kennel block to the assessment room. A single lead was attached to the wall for a 1.8 m leash to restrain the dog. During the assessment, one researcher acted as the handler, and a second person helped in observer interaction and implementing two tests requiring two people (Stranger and Fake toddler tests, described below). Data for all the following tests were recorded using a video recorder (Digital Video Recorder 1.1, Model: XQ-L400H, Manufacture: Kobi, Seoul, Korea).

##### Exploring the Room, One Minute

The handler entered the room, dropped the lead attached to the dog, and sat in the centre on a chair. Then, the observer started a timer and waited for 1 min without any interaction with the dog by either person.

##### Sociability to Handler

At the end of test 1, the handler called the dog to them in a friendly voice, remaining in the chair with no other body movement. If there was no response, a second attempt was made, and if still no response the handler clapped their hands on their lap and said ‘come here’ in the direction of the dog, trying at least three times to call the dog to them. When the dog came (at the first, second, or third call), the handler picked up the leash and then stroked the dog from the base of neck to tail three times. If the dog did not respond to the first, second, or third, call the handler approached the dog, picked up the leash, and gave the dog three strokes from the base of neck to tail. Following each stroke, the observer and handler counted 10 s, with behaviours exhibited noted.

##### Tolerance to Handling

The handler dropped the leash and held the dog’s collar. With the dog standing, the other handler (in the standing position, or crouching if a small breed of dog) picked up the dog’s rear inside foot, then the front inside foot, then reached over its back to pick up its rear outside foot, and finally the front outside foot. Each foot was held for 2 s. After picking up all four paws in this manner, the handler stood for 10 s with no dog interaction and finally removed the dog’s leash.

##### Toy Interactions

A tennis ball, squeaky toy, and tugging rope were shown to the dog and gently thrown across the room, and the handler verbally engaged the dog in play. If the dog picked up the ball, the handler waited to see if it returned to the handler without encouragement. If it did not, the handler encouraged the dog to bring the ball back by calling his/her name and saying “come”. If the dog still did not return, the handler went to the dog.

In both situations, the handler waited 10 s to see if the dog dropped the ball. If it did not, he/she asked the dog to “drop it”. If the dog did not respond, then a second command was given, “give”, and if necessary, a third attempt, “out”, was tried. If the dog did not respond to these commands, the handler approached the dog carefully and removed the ball from the dog’s mouth. These steps were repeated for a second throw, and after completion, the handler waited 10 s with no interaction before moving on to the next test.

##### Tag (Run and Freeze)

The run and freeze test was used to mimic a tag game. The handler gently moved the dog to the opposite end of the room and left it standing against the wall. Then, he gently moved one hand over its head, down toward the back to gently tap the rump area, and then ran across the room, laughing and waving arms, followed by suddenly stopping, folding his arms, and ignoring the dog. The tap, run, and freeze series was repeated a second time. The handler waited for 10 s after the run and freeze, ignoring the dog, before moving onto the next test. The dog was then placed back on the leash.

##### Resource Guarding

The handler tethered the dog to the wall for safety reasons, and proceeded to give the dog wet canned food, smeared in a bowl. The bowl was then placed near the dog at the end of the leash perimeter, allowing the dog to begin eating for 2 s. The handler then proceeded with a plastic hand, walking to the side of the dog while it was eating. Using the fake hand, the handler patted the dog on the head, continuing to stroke down its back and body twice. The fake hand was then placed 5 cm in front of the bowl and moved around in a semi-circle. The hand was then placed on the inside edge of the bowl and moved around the edge of the bowl next to the dog’s face, without touching it. Finally, the bowl was pulled away from the dog using the fake hand. The bowl was then returned to the dog, which was observed for 10 s.

The handler then gave the dog a pig’s ear or bone, depending on dog’s food interest, and it was allowed to chew it for 30 s. The steps above with wet food were repeated; then, the handler attempted to retrieve the food, asking the dog to “drop it”, “leave it”, or “give” before attempting to retrieve it by offering a higher value treat/food, e.g., the pig’s ear.

##### Stranger Interaction

The handler placed the dog on a leash as the observer exited the room and returned dressed in a reflective vest, large brimmed hat and using a walking stick. The observer entered the room, and bent down to extend an open flat hand as if to pat the dog on the head. The observer then talked to the dog normally and stopped for 3 s, allowing the dog to approach. If the dog approached, the observer patted the dog on the top of its head for 3 s. If the dog did not approach, it was observed for 10 s, with an emphasis on any interaction between the handler and/or the observer.

##### Fake Toddler Interaction

The handler stood and held the dog’s leash while the observer exited the area and returned carrying a toddler doll simulating a small child. Once the toddler was within the leash perimeter from the dog, the observer placed the doll on the floor facing the dog, with the doll’s arm extended toward the dog. The handler allowed the dog to approach if it desired. If the dog did not approach the observer, it was observed for 20 s.

##### Time Alone

The handler and observer removed the leash from the dog and left the room for 2 min, with a video camera in the front of the room monitoring behaviour and vocalisations. Then, the handler and observer re-entered through the same door.

##### Behaviour with Another Dog

This test was conducted in a yard (10−20 m), allowing adequate space between the test dog and another dog, both with handlers. Each dog had a handler, who interacted with their dog by giving treats and ignoring the other handler and dog. The handler had a short, 1 m, leash, so that the dog walked close to the handler. At the start, both handlers walked parallel to each other, 5 m apart, with the dogs on the outside. If one or both dogs were reactive and pulled toward each other, the distance between the handlers was increased. If both dogs were relaxed and focused on their handler, the handlers moved the dogs to an exercise circle. If the dogs did not breach a minimum distance of 5 m between them, they were introduced on opposite sides of a fence. There followed a circling activity, which required one handler to stand still with their dog on no more than 1.5 m of leash while the other handler and their dog completed a circle around the handler. Handlers then swapped places and repeated the circling activity. If no adverse behaviours were displayed, the handler in the middle of the circle remained at that location, ensuring that the only tension on the leash was from the dog. The other handler identified the leash threshold of the dog in the centre and moved close enough to allow the dogs to be nose to nose, also ensuring that the only tension on their leads was caused by the dog pulling, not them pulling against the dog. Once the leads became loose, and the dogs stopped pulling against the handler, the handlers took a step closer to each other, allowing the dogs to interact if they chose. Leashes remained loose. If there were signs of adverse reactions or aggression, dogs were then separated by increasing the threshold.

### 2.5. Behaviour Scoring

Following preliminary observation of dogs in their kennel and during the formal behaviour assessment, an ethogram with 48 behaviours, classified as either long duration behaviours (for which the duration was recorded) or events (for which the number of occurrences was recorded) was devised. The behaviours focused on eight components: activities of the mouth, body, tail, tail movement, ears, eyes, position, and movement (Table 1). Descriptions of each behaviour are presented in Table 2 and their connection to emotions (Anxiety, Fear, Friendliness, Arousal, Aggression) [22,23,24,25,26] in Table 3. Kennel behaviours were continuously recorded over a 1 h period (07:00–08:00), and the formal behaviour assessments were recorded for all tests. Behaviour recording was assisted by coding software (BORIS) [27]. The following behaviour variables with no or only one occurrence were discarded: Squint, Whale eyes.

The RSPCA staff classified the dogs for adoption suitability following the formal behaviour assessment: (1) pass and ready for adoption, (2) some behaviour issues which should be addressed in a behaviour modification program, and (3) fail due to extreme behaviour problems. However, in the current study no dogs were classified under category 2.

### 2.6. Statistical Analysis

Results were analysed using Minitab 17, Lead technology Inc., Pennsylvania State University, Pennsylvanina, USA. Behaviours were entered as the percentage of the total observation time or percentage of the frequency of occurrence during their period in the kennel and during the behaviour assessment. These two were compared using multivariate general linear models with the following factors: reason for surrender, age, weight, animals, days since entry, and outcome (adopted/euthanized). Residuals were checked for normal distribution using the Anderson Darling test. Spearman’s rank order correlations were computed between kennel and formal behaviour assessment variables. As comparisons with 38 other behaviours were made for each behaviour in each test of the behaviour assessment, results were corrected for false discovery using the Benjamini-Hochberg procedure [28]. The Bonferroni correction was rejected as it assumes independence in the individual tests. The Benjamini-Hochberg procedure ranks the P values for each test and compares P values to critical values [(rank/no. tests) x false discovery rate (selected as 0.20 as recommended by McDonald, 2014)]. All P values up to the critical one were considered to indicate a significant difference [28]. Correlations were further analysed on tests of the sample split according to owner surrender information, sex, adopted vs euthanasia, and daily behaviours. Linear and Binary Logistics Regressions were conducted to compare dog behaviour with RSPCA classification of outcomes and comparing behaviours over days for different tests. Two tests, Time Alone and Exploration of the Room, were subjected to additional logistic regression because of their predictive ability for kennel behaviour.

## 3. Results

### 3.1. Reasons for Dog Surrender

The reasons for surrender were moving away or insufficient time to care for the dog (22.2%); dog being aggressive or escaping, or family issues (8.3%); medical concerns (5.5%); and arousal, barking, chasing, destruction, owner’s death, resource guarding, or separation anxiety (2.8%).

### 3.2. Emotional Characteristics of Dogs in Their Kennels That Were or Were Not Subsequently Euthanased

#### 3.2.1. Emotional States of Dogs in Their Kennel

Over the first five days, dogs spent most time and had the highest frequencies of the following behaviours (Table 2): weight back, balanced body, and jumping up. Tail movement and position were spent in tail low and medium with still or slow movement, not wagging (Table 2). Ear position was most commonly ears back, then ears open, and finally ears forward. Eye direction was most commonly direct and diverting. In regards to position, dogs spent the most of the time in a kennel at the wire or front and the least amount of time in bed/sleeping or at the wall. Movement patterns were commonly standing, sit/lay, and pacing (Table 2). Over the five-day period, dogs spent 36% of their time in friendly behaviours, 25% displaying fear, 13% displaying anxiousness, 15% in high arousal, and 7% displaying aggression. Dogs’ frequency of emotions differed from duration, with 33% of occurrences being high arousal, 25% friendliness, 24% anxiousness, 16% fear, and 2% aggression. Thus, friendliness and fear were displayed less frequently but for a longer duration compared with arousal and anxiousness, which were of short duration but more frequent.

**Figure 1:**
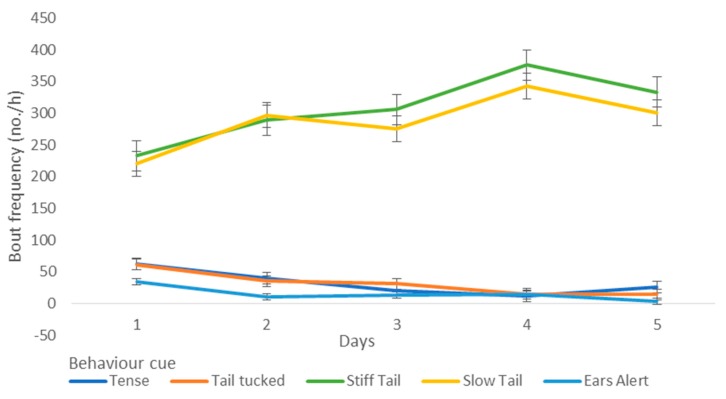
The frequency of fear-related behaviours, alert ears, and tail behaviours over the first five days that dogs (*n* = 38) spent in a shelter.

**Figure 2:**
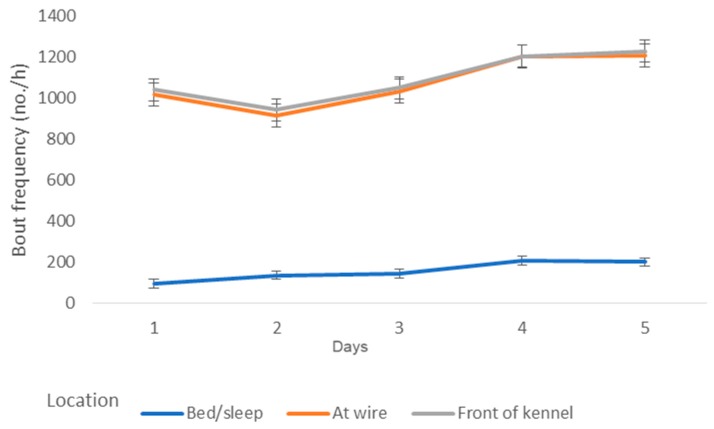
The frequency of position over the first five days that dogs (*n* = 38) spent in a shelter.

Over the five-day period, there was a significant reduction in the frequency of fear-related behaviours, including tense body posture (*p* < 0.05), tail tucked (*p* < 0.05), and alert response in ears (*p* < 0.05) (Figure 1). There was increases in stiff and slow tail movement (*p* < 0.05) (Figure 1) and the duration of time spent at the front of the kennel (*p* = 0.016), wire of the kennel (*p* = 0.008), and in bed/sleep (*p* = 0.0019) (Figure 1 and Figure 2).

**Figure 3:**
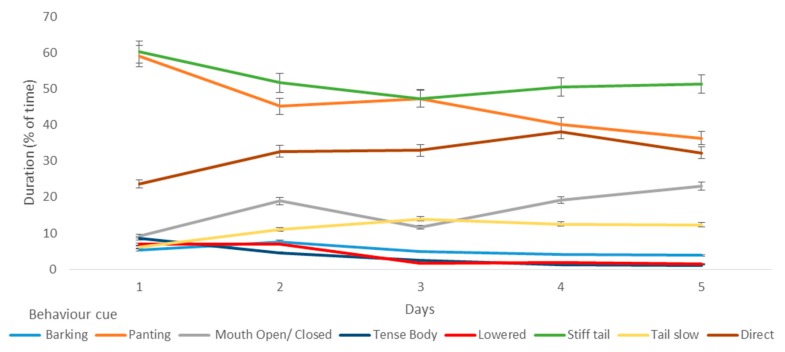
The duration of fear-related behaviours, arousal behaviours, and tail behaviours over the first five days that dogs (*n* = 38) spent in a shelter.

There were reductions in time spent panting (*p* < 0.001) (and corresponding increase in mouth open or closed, *p* < 0.001), a reduction in lowered (*p* < 0.008) and tense body posture (*p* < 0.001), and reductions in tucked tail and stiff tail movement, and a corresponding increase in slow tail movement (*p* < 0.05) (Figure 3).

#### 3.2.2. Relationship between Kennel Behaviour and Outcome for the Dogs 

**Figure 4:**
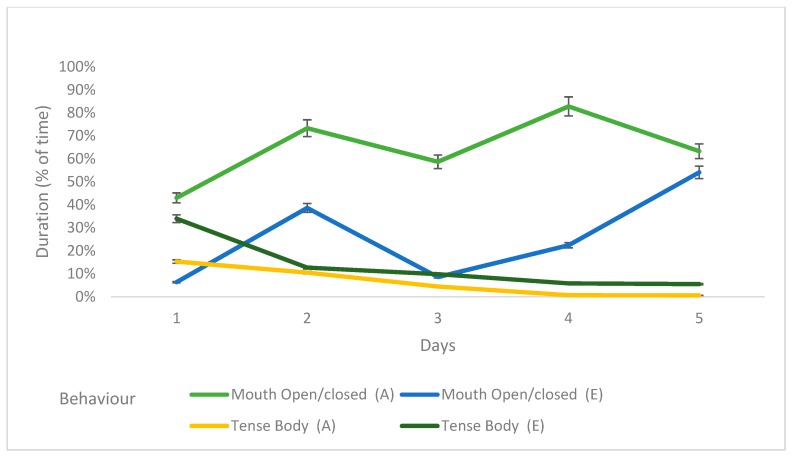
The duration of behaviours over the first five days that adopted or euthanased dogs (*n* = 38) spent in a shelter.

**Figure 5:**
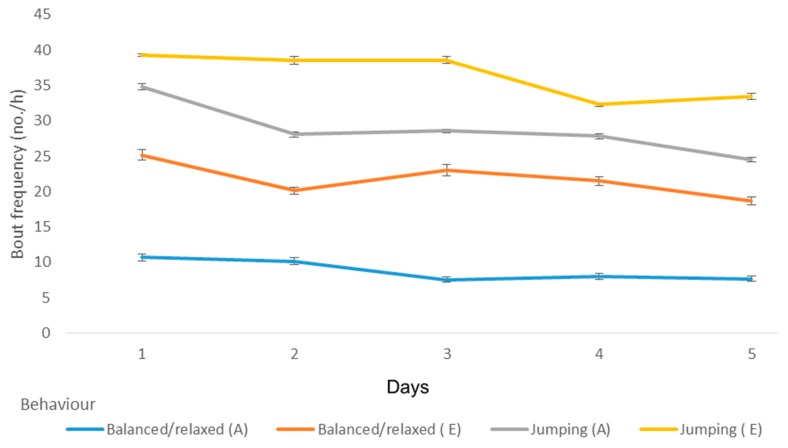
The frequency of behaviours over the first five days that adopted or euthanased dogs (*n* = 38) spent in a shelter.

Comparing behavioural characteristics of dogs that were adopted or euthanized, the latter had an increased duration of tense body posture overall, but inspection of changes over time revealed that this was mainly on the first day, with this behaviour declining over time in both sets of dogs (*p* = 0.001) (Table 4, Figure 4). Conversely, dogs that were adopted, which generally exhibited more mouth open/closed behaviour, had similar levels to euthanased dogs by day 5. Dogs that were adopted had a greater frequency of balanced/relaxed posture, but this declined over time, in contrast to euthanased dogs, which had little evidence of decline over time (*p* = 0.004). Jumping kennel was more common in euthanased dogs, and this declined over time in both euthanased and adopted dogs (*p* = 0.03) (Figure 5).

### 3.3. Emotional Characteristics of Dogs in the Behavioral Assessment That Were or Were Not Subsequently Euthanased

#### Behaviour of Dogs in Formal Behaviour Assessment

In the behaviour assessment, dogs spent 39% of their time in friendly behaviours, 17% displaying fear, 17% displaying anxiousness, 24% in high arousal, and 3% displaying aggression. Considering the frequency of behaviours, 26% were incidences of high arousal, 41% friendliness, 19% anxiousness, 12% fear, and 2% aggression.

Total scores for each behaviour were obtained from the formal behavioural assessment and categorised into emotional domains (Anxiety, Fear, Friendliness, Aggression, and Arousal). See Table 5 for Pearson’s correlations of scores, with significance levels corrected using the Benjamini-Hochberg procedure. 

Almost all correlations were statistically significant but ranged from weak to strong for both positive and negative correlations. There were positive correlations between the following behaviours that we associated with Fear: ears back, lip licking, lowered body, lowered head, shiver, tail low, tail tucked, tense body posture, weight back, and yawning; Anxiousness: fast, high tail, jumping, licking, lip licking, medium tail, pacing, panting, stiff tail, tense body posture, weight back, weight forward, and yawning. There were positive correlations between the following behaviours that we associated with Aggression: biting, ears forward, growling, high tail, lip licking, lowered head, medium tail, snapping, standing, stiff tail and still tail, and targeting gaze. There were positive correlations between the following behaviours that we associated with Arousal: barking, diverting gaze, fast and high tail, jumping up and off, licking, medium tail, mouthing, pacing, panting, weight forward, and whining. There were positive correlations between the following behaviours that we associated with Friendliness: balanced body posture, body curve, direct eye contact, ears forward and open, fast tail, handler interaction, jumping, medium tail, play behaviour, relaxed body, slow tail movement, sniffing, soft eye contact, wag loose, and walking.

### 3.4. Relationship between Kennel Behaviour and Formal Behaviour Assessment

There were positive correlations between anxiety, fear, and arousal behaviours displayed in kennels and in the formal behaviour assessment: whining, diverting eye contact, lip licking, panting, barking, jumping up, ears alert and forward, ears back, lowered body and tense body posture, tail tucked and stiff, and body weight back (*p* < 0.02) (Table 6). In addition, there were positive correlations between position in the kennel (at wall, wire, and at front door) and locations in behaviour assessment (at wall, window, and door) (*p* < 0.02) (Table 7).

#### 3.4.1. Exploration of Room

Comparing exploration of the room in the behaviour assessment with kennel behaviours, there were significant correlations between many duration and frequency behaviours in the anxiety, arousal, and fear emotional states (Table 8). Nearly all correlations were positive, demonstrating that for most behaviours recorded in the kennel were related to those exhibited in the behavioural assessment. Only two—whining and lip licking—were negatively related, suggesting that these are not reliable indicators of the room exploration test.

#### 3.4.2. Time Alone Assessment

Similarly, comparing the time alone assessment with kennel behaviours, there were also significant correlations between many duration and frequency behaviours in the anxiety, arousal and fear emotional states (Table 9). Nearly all correlations were positive, demonstrating that most behaviours recorded in the kennel were related to those exhibited in the time alone assessment. Only three—whining, fast tail, and direct eyes—were negatively related, suggesting that these are not reliable indicators of the time alone test. There were also positive correlations between locations (Table 10).

#### 3.4.3. Relationship between Outcomes for the Dogs and Summarised Behaviour Results

Comparing the time spent in the various behaviours for dogs that were adopted with those that were euthanased, dogs that displayed more barking, balanced or lowered posture, or positioned by the wall in the kennel assessment, or balanced/lowered posture or pacing in the behaviour assessment, or balanced posture or jumping up in the time alone test had an increased likelihood of being adopted (Table 11). Those that displayed more tense body posture in the kennel test or sitting/lying in the behavioural assessment were more likely to be euthanased.

Comparing the frequency of the various behaviours for dogs that were adopted with those that were euthanased, dogs that displayed more barking in the kennel assessment or balanced posture in the kennel or behaviour assessment or the time alone test had an increased likelihood of being adopted (Table 12). Those that displayed more panting in the kennel assessment, lowered head, or scanning in the behaviour assessment were more likely to be euthanased.

## 4. Discussion

One solution to increasing adoptability of shelter dogs is the early detection of behaviour problems followed by modification programs aimed at helping dogs develop desired behaviours. Longitudinal monitoring of behaviours using both kennel and formal behaviour assessment information to help create comprehensive insight of the dog’s behaviour can help achieve this aim [20]. Recent studies have pointed to the uncertainty of single behaviour assessments [16], but the work of Goold and Newberry and this current research clearly demonstrate the benefit of continual monitoring. Continual monitoring allows correct identification of behavioural cues associated with separation-related behaviours, anxiety, fear, arousal, and friendliness. To identify these behavioural cues using monitoring tools in the first five days allows behaviour modification to be implemented to help these dogs to cope effectively in a socially isolating environment. Using a formal behaviour assessment, as customarily practiced in shelters, as a single context assessment of a dog’s behaviour creates an ineffective profile of stable behavioural tendencies.

### 4.1. Behaviour in the Five Days after Surrender

This study focused on behaviour observations in the first five days after admission to a shelter and compared these to behaviour identified in a formal behaviour assessment. Over the first five days after admission, dogs displayed decreasing tense body and tucked tail, which are probably the best indicators of fear in the dogs. Previous studies that found that over the first five days after relinquishment to a shelter dogs will experience social isolation due to the breaking of social bonds with previous companions/owners [5,6]. Prior studies report numerous contradictory indications of the extent to which shelter dogs adapt over time, displaying behavioural and physiological indicators of positive and negative stress [29]. Some studies report a reduction in stress and fear related behaviours over time in shelters [6,10,30], whereas others indicate that dogs display acute signs of negative stress and fear due to the high novelty of the shelter environment [29,31]. Although environmental factors influence these behaviours, including new olfactory, auditory, and sensory stimulation, dogs can either have a positive or negative coping style, thereby demonstrating effective or ineffective ability to cope in a new environment [21,29,32]. These diverse results are likely to be due to differences in resources offered by shelters.

The ability to monitor kennel behaviours associated with positive and negative stress or coping styles can help identify changes in the quality of life (QoL) of dogs in shelters [15,33]. Identifying dogs that have a deterioration in positive behaviours allows early treatment. Interestingly, dogs that were deemed not suitable for adoption had higher durations of tense body posture in-kennel and increased frequency of jumping behaviour in kennel. Conversely, positive behaviours, including a balanced/relaxed body posture, had lower frequency of occurrence in dogs suitable for adoption.

Another interesting finding in the present study is the association between positive behaviours that include friendliness in dogs in the first five days, which agrees with previous studies [6,10,20,30]. These findings highlight the benefit of longitudinal monitoring of behaviour in shelter kennels to identify stable behaviours that included docility and friendliness [20].

### 4.2. Behaviours in Assessment

Anxiousness, arousal, and fear tendencies correlated with its corresponding emotional domain in the behaviour assessment (Table 5), indicating a positive relationship with the domains identified in kennel and behaviour in the standardized assessment. Previous research by Mornement [26] in behaviour assessments in Australian shelters indicated fear and friendliness were the only behaviours that were predictive. Other research using similar test protocols with social (stranger and toddler interactions) and non-social stimuli reported fear related behaviours as found in this research [34,35]. As stated previously, the effect of acute stress and social isolation in dogs when relinquished to a novel environment have the ability to dramatically change behaviour. Thus, the result of increased fear, arousal, and anxious behaviour found in the kennel and at assessment (Table 4) suggest time-independent coping mechanisms that a dog may implement to help respond to the changing environment [21,36,37]. The results go beyond the previous study, suggesting that if coping mechanisms are ineffective at helping the dog cope with the environment, then those behavioural tendencies can manifest into behaviour problems that can be identified in an assessment.

### 4.3. Comparison between Kennel and Behaviour Assessment

The comparison of kennel behaviour and the formal behaviour assessment indicates that kennel behavioural cues associated with fear, anxiety, and arousal were confirmed in the formal behaviour assessment (Table 6). Furthermore, in the analysis of the position in kennel, we confirmed that position in the behaviour assessment was associated with front of kennel, door, and wall in each situation (Table 7).

Once the formal assessment was separated into component parts, specifically exploration of room and time alone, there were associations between behaviours found in these tests and kennel behaviours reflecting separation-related behavioural cues, anxiousness, arousal, and fear (Table 8 and Table 9). Separation related behaviours are associated with increased whining, pacing, excessive salivation, barking, jumping in orientation of owner’s departure, and escaping behaviour [38]. Studies show that separation-related behaviours can be correctly identified in video analysis of dogs in their time alone once the owner has left [18]. Furthermore, a study by Blackwell et al. [39] into the identification of separation-related behaviours in shelters showed the importance of using a time-alone test to assess dogs with behaviour problems. The results clearly demonstrate the positive predictive value of the time alone test to identify separation related behaviours [39]. Separation-related behaviours have been identified as a common problem post adoption [13]. Therefore, to identify these issues early is the key to early treatment, which could lead to an increase in the likelihood of successful adoption and therefore decreasing euthanasia. The findings with respect to fear are consistent with that of Mornement [26], who identified its predictive validity. Research by Tiira et al. [40] outlined high comorbidity between different anxieties, showing that fearful dogs had significantly higher noise sensitivity and separation anxiety.

Dogs with behaviours associated with separation-related problems, such as arousal and fear, were less likely to be deemed adoptable (Table 11 and Table 12). Dogs that displayed friendly, low arousal, and docile behaviours were more likely to be adopted (Table 11 and Table 12). Behavioural issues that have been linked to reasons for relinquishment of dogs include separation-related behaviours, arousal, and fear [41,42,43,44,45,46]. In contrast, behaviours that adoptees look for in dogs are associated with friendliness toward people, docility, and low arousal [47]. Thus, increasing positive behaviours and decreasing separation-related behaviours, fear, and high arousal are critical to increase adoptability, thereby decreasing euthanasia. Early recognition of ineffective behaviours and coping mechanisms allows shelters to implement behaviour management programs before behavioural problems manifest [48,49]. Behaviour assessments are comprised of numerous tests that allow for a snapshot of a dog’s behaviour that is multifactorial. Therefore, a paradigm shift should occur in shelters to implement assessments as continuous tools to monitor a dogs’ behaviour over time. Once unsuitable or problem behaviours are identified, shelters can create effective modification plans to allow issues to be solved before manifesting into serious behavioural problems. Using assessments in shelters to identify past behaviours in the previous home or to predict future behaviour is difficult. However, using assessments as a tool to understand the behaviour of dogs in conjunction with continual kennel monitoring and everyday interaction may allow identification of behavioural issues and ineffective coping mechanisms. Further research into monitoring of behaviours associated with the manifestation of behavioural problems in shelters is warranted.

Some limitations are associated with this research that future studies should consider. To allow for comprehensive behaviour analysis of dogs, previous home environment could be taken into consideration. Therefore, we should try to more accurately represent behaviour in the home. Our sample size was relatively small, but due to the nature of the study, which identified changes in behaviours over time on single dogs, it is not seen as a major restriction. Finally, the limitation of variability between each shelter should be taken into consideration and warrants further study.

## 5. Conclusions

Previous research suggests that behaviour assessments are ineffective, focusing on the lack of their accurate predictability of behaviour. However, in this study, we found that behaviour assessment information can be related to behaviour over the previous days since relinquishment to the novel environment. Effectively monitoring kennel behaviour allows early recognition of problems. Numerous authors have recommended continual monitoring procedures to help identify key behavioural problems as early as possible. This research has demonstrated numerous correlations between kennel behaviour and that displayed during formal assessments. We suggest that shelters should use continuous monitoring techniques at the same time as supporting automated behaviour problem recognition. Continuing to use formal assessments and incorporating longitudinal monitoring of behaviour to help identify dogs unable to cope effectively in shelter environments may also provide useful additional information of dog behaviour problems. Such monitoring allows early implementation of training modification, thereby increasing adoptability of dogs that once would be deemed unadoptable.

## Figures and Tables

**Table 1:** Canine behaviours recorded for each body part, as well as positions and movement types.

Mouth	Body	Tail	Tail Movement	Ears	Eyes	Position	Movement
Open/Closed	Weight forward	Low	Wagging	Alert	Soft	Front	Pacing
Panting	Weight back	Med	Fast	Back	Hard	Bed/Sleep	Sit/Lay
Mouthing	Balanced	High	Stiff	Forward	Direct	Wire	Stand
Lip Lick	Relaxed	Tucked	Slow	Open	Squinty	Wall	Still
Snap	Tense				Whale Eyes		
Bite	Lowered				Dilated		
Whining	Play bow				Targeting		
Barking	Jumping up				Diverting		
Growl	Lowered head						
Howling	piloerect						

**Table 2:** Behaviours measured, their descriptions and mean values (± SEM) for duration and frequency during kennel observations.

Behaviour	Description	Duration (s/days)	Frequency (no./days)
Mouth			
Open/Closed	Mouth is open or close, no visual signs of panting	3017 ± 568.0	4.35 ± 0.83
Panting	Increased respiration, deep gasps, and salivation	8314 ± 654.0	22.2 ± 5.35
Mouthing	Nipping or play biting	0	0
Lip Lick	Licking of the upper lip	21.9 ± 21.60	0.17 ± 0.040
Snap	Rapid open and close mouth, possible baring teeth, growl, bark, lunge	0	0
Bite	Closure the teeth on victim causing a wound	0	0
Whining	A sustained, high pitched, plaintive sound	0.464 ± 0.360	0.10 ± 0.060
Barking	Brief vocalization	952 ± 299.0	19.2 ± 5.86
Growl	Low guttural prolonged vocalisation	6.99 ± 6.990	0.12 ± 0.060
Howling	Raise muzzle perpendicular to ground, vocalise over extended period, open jaws	0	0
Body			
Weight forward	Body weight forward while standing still	204 ± 82.7	4.5 ± 2.03
Weight back	Body weight back while standing still	3371 ± 466.0	17.7± 2.823
Balanced	Balanced body posture standing still	6546 ± 633.0	37.6 ±4.960
Relaxed	Body posture relaxed in movement	0	0
Tense	Body Posture is still and tense in association to stimulus	669 ± 147.2	3.98 ±0.922
Lowered	Body lowered to ground	728 ± 183.1	4.85 ±1.1676
Play bow	Stretching front legs out in front, leaning down on its elbows	14.1 ± 11.70	0.625 ±0.2454
Jumping up	Jumping in air	568 ± 115.1	31.9 ± 5.21
Lowered head	Lowered head as body posture is high	0	0
Piloerect	Hackles rise	0	0
Tail			
Low	Tail positioned low	6438 ± 469.6	25.4 ±3.56
Med	Tail positioned in line with spine	2945 ± 404.7	23.6 ± 4.34
High	Tail high or above spine	871 ± 325.2	6.85 ± 1.86
Tucked	Tail positioned underneath body	1721 ± 395.1	3.91 ± 0.6460
Tail Movement			
Wagging	Relaxed tail movement	0	0
Fast	Movement of tail fast	29.8 ± 16.10	1.88 ± 1.2500
Stiff	Still and no movement in tail	9531 ± 397.1	38.4 ± 3.68
Slow	Slow movement of the tail	2030 + 264.1	35.9 +- 4.17
Ears			
Alert	Ears forward and directed at an object, stimulus, or sound	243 + 169.0	1.88 + 0.8610
Back	Ears positioned back and flat	4470 + 617.0	18.0 + 3.07
Forward	Ears positioned forward	3066 ± 621.0	9.6 ± 1.760
Open	Ears neutral	4221 ± 473.0	17.0 ± 2.22
Eyes			
Soft	Relaxed eyes	275.6 ± 80.5	1.85 ± 0.361
Hard	Hard focused stare	0	0
Direct	Directed at object	5832 ± 516.0	11.4 ±- 0.997
Squinting	Eyes not fully open	0	0
Whale Eyes	Showing whites of eyes	0	0
Dilated	Pupil dilation	219 ± 137.0	1.28 ± 0.699
Targeting	Constricted pupils and targeting object or stimulus	0	0
Diverting	Eyes moving and not maintaining eye contact	5585 ± 484.0	11.1 ± 0.94
Position			
Front	At the front of the kennel/front of room	4705 ± 388.0	136.9 ± 12.70
Bed/Sleep	In bed	1022 ± 224.0	19.8 ± 3.69
Wire	At wire	5065 ± 334.0	134.8 ± 12.50
Wall	At wall of kennel or in behaviour assessment room	1303 ± 237.0	18.7 ± 2.46
Movement			
Pacing	Repeated movement in a regular pattern	3540 ± 308.0	128.8 ± 9.62
Sit/Lie	Sitting position	4290 ± 352.0	62.4 ± 5.19
Stand	Standing on all fours	4242 ± 295.0	119.5 ± 9.45
Still	Motionless	0	0
Walking	Progressive locomotion with at least three legs on floor at one time	0	0

**Table 3:** The behaviours contributing to the emotional states Fear, Anxiety, Aggression, Arousal, and Friendliness.

Behaviour Number															
1	2	3	4	5	6	7	8	9	10	11	12	13	14	15	16
**Fear**															
Diverting	Ears Back	Lip Licking	Lowered Body	Lowered head	Shiver	stiff tail	Tail Low	Tail tucked	Tense Body Posture	Weight back	Yawn				
**Anxiety**															
Fast tail	High tail	Jumping	Licking	Lip licking	Medium	Pacing	Panting	Stiff Tail	Tense body	Weight back	Weight forward	Whining			
**Aggression**															
Biting	Ears Forward	Growling	High tail	Lip Licking	Lowered head	Mediuam tail	Snapping	Standing	Stiff tail	Still tail	Targetting	Vertical Lip Raise			
**Arousal**															
Barking	Diverting Gaze	Fast tail	High Tail	Jumping up	Jump off	Licking	Medium Tail	Mouthing	Pacing	Panting	Weight forward	whining			
**Arousal**															
Balanced	Body Curve	Direct eye	Ears forward	Ears open	Fast tail	Handler interaction	Jump	Medium Tail	Play	Relaxed body	slow	Sniff	Soft	Tail loose	Walking

**Table 4:** Differences in kennel behaviour between dogs that were euthanased and adopted, either overall or on certain days.

F/D	Behaviours	Interaction	*p*-Value
F	Tense body	A/E	0.001
D	Balance/relaxed	A/E	0.002
D	Tense body	A/E	0.002

D = Duration, F = Frequency; A/E = Adopted/Euthanased.

**Table 5:** Spearman Rank correlation coefficients between behaviours recorded in the formal behaviour assessment in shelter dogs (*n* = 38) (Numbered behaviours relate to those presented in Table 3).

	**Fear**																
		**1**	**2**	**3**	**4**	**5**	**6**	**7**	**8**	**9**	**10**	**11**	**12**				
**Fear**	**1**		*0.40*	*0.32*	−0.22	*0.33*	0.19	*0.40*	0.17	0.27	−0.11	0.09	−0.18				
	**2**			**0.61**	**0.48**	**0.60**	−0.26	−0.03	0.21	0.02	**0.49**	**0.43**	*0.38*				
	**3**				0.02	0.02	0.12	*0.35*	*−0.35*	−0.10	0.31	0.19	0.00				
	**4**					0.08	−0.18	**0.55**	*0.32*	**0.56**	−0.06	**0.61**	0.00				
	**5**						−0.18	−0.04	0.03	*0.34*	*0.36*	*0.32*	0.00				
	**6**							0.32	0.15	−0.08	0.21	−0.22	0.00				
	**7**								**0.41**	**0.60**	*0.48*	**0.59**	0.00				
	**8**									**0.65**	0.18	**0.45**	0.00				
	**9**										**0.43**	**0.42**	0.00				
	**10**											−0.15	**0.54**				
	**11**												0.00				
	**Anxiety**																
**Anxiety**		**1**	**2**	**3**	**4**	**5**	**6**	**7**	**8**	**9**	**10**	**11**	**12**	**13**	**14**		
	**1**		0.18	**0.51**	*0.35*	−0.05	**0.77**	0.32	**0.47**	**−0.64**	−0.13	*−0.32*	**0.42**	−0.03	*0.36*		
	**2**			−0.11	−0.14	0.00	*−0.35*	−0.20	−0.20	−0.17	−0.14	−0.17	*0.41*	0.17	0.00		
	**3**				−0.12	−0.08	*0.37*	**0.40**	**0.39**	−0.16	−0.12	−0.08	**0.53**	0.15	0.00		
	**4**					−0.01	−0.20	**0.56**	**0.36**	−0.02	**0.69**	−0.07	**0.64**	**0.39**	0.00		
	**5**						−0.05	0.13	**0.56**	**0.44**	**0.63**	0.19	**0.81**	−0.14	0.00		
	**6**							**0.53**	**0.37**	**−0.37**	0.07	−0.07	**0.38**	−0.23	0.00		
	**7**								0.56	**0.44**	**0.63**	**0.48**	**0.47**	0.09	0.00		
	**8**									0.04	−0.01	0.05	**0.47**	−0.07	0.00		
	**9**										**0.48**	**0.59**	−0.29	−0.16	0.00		
	**10**											−0.15	**0.72**	**0.43**	**0.53**		
	**11**												*0.35*	0.22	0.00		
	**12**													0.03	**0.49**		
	**13**														0.00		
	**Aggression**																
**Aggression**		**1**	**2**	**3**	**4**	**5**	**6**	**7**	**8**	**9**	**10**	**11**	**12**	**13**			
	**1**		*0.33*	**0.90**	−0.08	*0.31*	0.24	−0.01	**0.72**	0.07	*0.29*	**0.79**	−0.03	0.00			
	**2**			0.25	0.21	−0.19	−0.16	−0.15	0.18	0.16	0.12	0.16	−0.11	*0.33*			
	**3**				−0.03	−0.01	**0.55**	*0.34*	**0.97**	**0.59**	0.05	**0.71**	−0.04	**0.90**			
	**4**					−0.33	**−0.37**	−0.19	−0.01	0.04	0.08	0.22	**0.44**	−0.08			
	**5**						*0.31*	0.16	−0.08	−0.02	0.35	0.27	0.10	*0.31*			
	**6**							0.24	**0.51**	**0.41**	0.13	0.06	−0.17	0.24			
	**7**								*0.36*	**0.44**	*−0.37*	0.02	0.05	−0.01			
	**8**									**0.60**	**0.55**	−0.02	**0.72**	0.01			
	**9**										−0.02	0.06	0.07	0.07			
	**10**											*0.32*	0.24	*0.29*			
	**11**												**0.70**	**0.70**			
	**12**													−0.03			
	**Arousal**																
**Arousal**		**1**	**2**	**3**	**4**	**5**	**6**	**7**	**8**	**9**	**10**	**11**	**12**	**13**			
	**1**		0.05	−0.23	**0.45**	−0.10	−0.09	−0.20	−0.24	−0.19	−0.19	−0.11	−0.09	−0.16			
	**2**			*0.35*	0.27	*0.33*	−0.03	*0.31*	0.26	*0.34*	0.23	**0.37**	*0.31*	0.03			
	**3**				−0.08	**0.51**	0.09	*0.32*	**0.77**	*0.32*	0.24	**0.47**	**0.41**	−0.16			
	**4**					−0.06	−0.07	*0.33*	−0.19	−0.03	0.26	0.06	0.20	0.02			
	**5**						−0.02	0.02	*0.37*	**0.43**	**0.40**	**0.38**	**0.52**	0.13			
	**6**							0.09	−0.02	−0.15	**0.46**	**0.41**	0.15	−0.16			
	**7**								0.16	**0.44**	**0.56**	**0.36**	**0.64**	**0.39**			
	**8**									**0.51**	0.16	*0.37*	**0.38**	−0.01			
	**9**										0.15	*0.34*	**0.42**	*0.31*			
	**10**											**0.56**	**0.81**	0.10			
	**11**												**0.47**	−0.21			
	**12**													*0.30*			
	**Friendliness**																
**Friendliness**		**1**	**2**	**3**	**4**	**5**	**6**	**7**	**8**	**9**	**10**	**11**	**12**	**13**	**14**	**15**	**16**
	**1**		−0.03	0.30	**0.39**	**0.36**	0.08	−0.16	*−0.31*	0.18	0.18	**−0.47**	**0.54**	0.05	−0.04	0.11	**0.51**
	**2**			**0.36**	−0.10	−0.17	*0.36*	*0.31*	0.20	*0.35*	0.03	*−0.36*	0.15	**−0.39**	**−0.47**	−0.25	0.13
	**3**				0.09	0.01	*0.35*	0.14	−0.22	**0.39**	−0.01	−0.15	0.26	−0.18	**−0.40**	−0.10	0.08
	**4**					**−0.54**	*−0.37*	−0.01	−0.28	−0.15	0.26	0.11	0.20	*0.36*	0.03	0.14	0.01
	**5**						0.21	*−0.06*	0.08	0.11	*0.31*	0.09	0.17	0.25	0.15	0.06	0.24
	**6**							0.30	**0.51**	**0.77**	−0.04	−0.25	0.04	−0.25	−0.25	−0.12	0.02
	**7**								*0.30*	0.32	0.10	*0.33*	0.00	−0.20	0.25	0.12	−0.24
	**8**									*0.37*	**0.43**	−0.24	−0.18	*0.39*	**0.38**	−0.15	−0.08
	**9**										0.07	−0.13	0.27	−0.21	−0.14	−0.01	−0.08
	**10**											**0.53**	0.21	**0.48**	*0.34*	−0.04	−0.03
	**11**												0.05	*0.36*	**0.39**	−0.06	−0.21
	**12**													−0.18	−0.19	0.04	**0.44**
	**13**														**0.47**	0.20	−0.05
	**14**															**0.65**	−0.24
	**15**																−0.01

**Table 6:** Significant (*p* < 0.05) Spearman Rank correlation coefficients between behaviours recorded in kennel and the formal behaviour assessment of shelter dogs (*n* = 38), listed for the emotional states of Arousal, Fear and Anxiety.

**Arousal**		**Fear**		**Anxiety**	
Barking	**0.57**	Diverting	*0.34*	Ears back	**0.57**
Diverting Gaze	*0.30*	Ears Back	**0.46**	Fast tail	**0.40**
Fast tail	**0.40**	Lip Licking	**0.42**	High tail	**0.63**
High Tail	**0.63**	Lowered Body	**0.44**	Jumping	*0.35*
Jumping up	**0.53**	Lowered head	**0.45**	Licking	*0.31*
Jump off	*0.35*	Shiver	**0.41**	Lip licking	*0.29*
Licking	*0.31*	Stiff tail	*0.33*	Medium tail	**0.45**
Lip licking	*0.29*	Tail Low	**0.45**	Pacing	*0.42*
Medium Tail	**0.45**	Tail tucked	*0.25*	Panting	*0.25*
Mouthing	*0.59*	Tense Body Posture	*0.28*	Stiff tail	*0.33*
Pacing	*0.31*	Weight back	**0.41**	Tense body	0.28
Panting	**0.42**	Yawn	*0.33*	Weight back	**0.41**
Weight forward	**0.38**			Weight forward	*0.38*
Whining	**0.36**			Whining	*0.36*

***p* < 0.01**, *p* < 0.05.

**Table 7:** Significant (*p* < 0.05) Spearman Rank correlation coefficients between locations recorded in kennel and formal behaviour assessment of shelter dogs (*n* = 38).

Location	Behaviour Assessment			
Kennel assessment	Door	Front of room	Wall	Window
Front of kennel	**0.45**	−0.08	−*0.36*	−0.11
Wall	−0.22	0.00	**0.49**	−0.23

***p* < 0.01**, *p <* 0.05

**Table 8:** Spearman Rank correlation coefficients between behaviours recorded in kennel and behaviours exhibited during the ‘exploration of room test’ in the behaviour assessment of shelter dogs (*n* = 38) within the emotional domains of arousal, fear, and anxiety.

Arousal	Title	Fear	Title	Anxiety	Title
Barking	*0.40*	Ears Back	**0.59**	Ears back	**0.59**
Diverting Gaze	*0.35*	Lip Licking	*−0.12*	Fast tail	**0.38**
High Tail	**0.69**	Lowered Body	*0.33*	High tail	**0.59**
Jumping up	**0.45**	Lowered head	**0.46**	Jumping	**0.28**
Jump off	*0.33*	Shiver	**0.52**	Licking	*0.34*
Licking	*0.35*	Stiff tail	**0.39**	Medium tail	*0.36*
Lip licking	0.27	*Tail Low*	*0.25*	Pacing	**0.44**
Medium Tail	*0.36*	Tail tucked	*0.25*	Panting	**0.46**
Pacing	*0.25*	Tense Body	*0.30*	Stiff tail	**0.39**
Panting	**0.46**	Weight back	**0.42**	Tense body	**0.36**
Weight forward	*0.33*			Weight back	**0.49**
Whining	*−0.50*				

***p* < 0.01**, *p* < *0.05.*

**Table 9:** Significant (*p* < 0.05) Spearman Rank correlation coefficients between behaviours recorded in kennel and behaviours exhibited during the time alone test in the behaviour assessment of shelter dogs (*n* = 38) within the emotional domains of arousal, fear, anxiety, and friendliness.

Arousal		Fear		Anxiety		Friendliness	
Barking	**0.54**	Diverting	**0.52**	Ears back	**0.64**	Direct eye	*−0.32*
Fast tail	*−0.24*	Ears Back	**0.45**	Fast tail	*0.36*	Ears forward	**0.61**
High Tail	**0.61**	Lowered Body	*0.28*	High tail	**0.61**	Ears open	*0.29*
Jumping up	**0.38**	Lowered head	*0.30*	Jumping	**0.38**	Fast tail	**0.50**
Licking	**0.47**	Tail Low	**0.42**	Lip licking	*0.28*	Mouth open	**0.48**
Lip licking	**0.46**	Tail tucked	**0.43**	Medium tail	**0.46**	Medium tail	**0.46**
Medium Tail	**0.43**	Tense Body Posture	*0.23*	Pacing	*0.36*	Relaxed body	**0.51**
Pacing	**0.41**	Weight back	**0.47**	Panting	**0.41**	slow	**0.52**
Weight forward	*0.31*			Stiff tail	**0.50**	Sniff	*0.35*
Whining	*−0.23*			Tense body	*0.33*	Stand	**0.51**
				Weight back	**0.47**	Walking	**0.39**
				Weight forward	*0.31*		
				Whining	*−0.23*		

***p* < 0.01**, *p* < *0.05.*

**Table 10:** Significant (*p* < 0.05) Spearman Rank correlation coefficients between locations recorded in kennel and the time alone test of shelter dogs (*n* = 38).

Kennel Assessment	Time Alone Test		
	Door	Wall	Window
Front	*0.34*		
Wall		**0.54**	
Window		**−0.45**	*0.31*

***p* < 0.01**, *p* < *0.05.*

**Table 11:** Time spent in behaviours in the kennel, the formal assessment and time alone test of dogs (*n* = 38) that were adopted or euthanased, with Odds Ratio and Confidence Interval (CI) tested by binary logistic regression.

K/B/T	Behaviour	Adopted (% time)	Euthanased (% time)	Odds Ratio	95% CI
K	Barking	5.58	1.30	**1.47**	0.98–2.21
K	Balanced	44.06	22.34	**1.23**	1.03–1.49
K	Lowered	3.25	4.30	**4.22**	0.98–18.16
K	Tense	1.50	6.49	**0.09**	0.01–1.07
K	Wall	7.99	6.60	*1.53*	0.96–2.42
K	Sit/Lay	21.51	24.93	**1.52**	0.44–1.00
B	Balanced	66.41	44.60	**1.67**	0.97–2.87
B	Lowered	7.97	16.04	**1.72**	0.84–3.48
B	Pacing	37.59	30.94	**1.58**	0.98–2.51
T	Panting	59.21	68.99	*0.95*	0.89–1.00
T	Balanced	78.67	44.66	**1.50**	1.10–2.04
T	Jump up	18.35	30.21	**1.44**	1.07–1.92

***p* < 0.01**, *p* < *0.05*. K: Kennel B: Behaviour assessment T: Time alone.

**Table 12:** Frequency of behaviours in the kennel, behaviours in the formal assessment and time alone test of dogs (*n* = 38) that were adopted or euthanased, together with the significance of the difference tested by binary logistic regression.

K/B/T	Behaviour	Adopted (% Frequency)	Euthanased (% Frequency)	Odds Ratio	95% CI
K	Barking	19.32	8.50	*1.09*	1.01–1.19
K	Panting	54.64	65.46	*0.95*	0.91–1.00
K	Balanced	45.24	26.13	**1.19**	1.05–1.35
B	Balanced	40.89	27.72	**1.48**	1.11–1.98
B	Lowered Head	12.29	12.77	**1.25**	0.99–1.55
B	Scanning	3.08	4.66	**0.65**	0.44–0.95
T	Balanced	54.68	35.58	**1.34**	1.11–1.62

***p* < 0.01**, *p* < *0.05*; K: Kennel B: Behaviour assessment T: Time alone.
