# Erratum: Clay, L.; Paterson, M.; Bennett, P.; Perry, G.; Phillips, C. Early Recognition of Behaviour Problems in Shelter Dogs by Monitoring Them in Their Kennels after Admission to a Shelter. *Animals* 2019, *9*, 875

**DOI:** 10.3390/ani9121150

**Published:** 2019-12-15

**Authors:** Liam Clay, Mandy Paterson, Pauleen Bennett, Gaille Perry, Clive Phillips

**Affiliations:** 1 Centre for Animal Welfare and Ethics, University of Queensland, Gatton, Queensland 4343, Australia; mpaterson@rspcaqld.org.au (M.P.); c.phillips@uq.edu.au (C.P.); 2 Royal Society for the Prevention of Cruelty to Animals Queensland, Brisbane, Queensland 4076, Australia; 3 School of Psychology and Public Health, La Trobe University, Bendigo, Victoria 3552, Australia; pauleen.bennett@latrobe.edu.au; 4 Delta Society, Summer Hill, New South Wales 2130, Australia; perrygaille@gmail.com

The authors wish to make the following corrections to their paper [1]:

In Table 3, arousal was repeated as a row heading at the end—it should have read “friendliness” (see Table 3 below).

In Table 4, there were errors in the behaviours, their method of recording, the interactions and p-values. The new Table 4 is given below. 

Scientific results were correctly stated in the text but incorrect in these two tables. The authors would like to apologize for any inconvenience caused.

## Figures and Tables

**Table 3:** The behaviours contributing to the emotional states of fear, anxiety, aggression, arousal, and friendliness.

Behaviour Number															
1	2	3	4	5	6	7	8	9	10	11	12	13	14	15	16
**Fear**															
Diverting	Ears Back	Lip Licking	Lowered Body	Lowered Head	Shiver	Stiff Tail	Tail Low	Tail Tucked	Tense Body Posture	Weight back	Yawn				
**Anxiety**															
Fast tail	High tail	Jumping	Licking	Lip licking	Medium	Pacing	Panting	Stiff Tail	Tense body	Weight Back	Weight Forward	Whining			
**Aggression**															
Biting	Ears Forward	Growling	High Tail	Lip Licking	Lowered Head	Medium Tail	Snapping	Standing	Stiff tail	Still tail	Targeting	Vertical Lip Raise			
**Arousal**															
Barking	Diverting Gaze	Fast Tail	High Tail	Jumping Up	Jump Off	Licking	Medium Tail	Mouthing	Pacing	Panting	Weight Forward	Whining			
**Friendliness**															
Balanced	Body Curve	Direct eye	Ears Forward	Ears Open	Fast Tail	Handler Interaction	Jump	Medium Tail	Play	Relaxed body	slow	Sniff	Soft	Tail Loose	Walking

**Table 4:** Differences in kennel behaviour between dogs that were euthanased and adopted, either overall or on certain days.

F/D	Behaviours	Interaction	*p*-Value
D	Tense body	Day 1	0.001
F	Balance/relaxed	Overall	0.004
F	Jumping in kennel	Overall	0.03

D = Duration, F = Frequency.
